# Prenatal bonding and early emotion regulation in infancy and toddlerhood (0–36 months): a systematic review of developmental associations, psychological mediators, and contextual moderators

**DOI:** 10.3389/fpsyg.2025.1700636

**Published:** 2025-11-12

**Authors:** Brenda Cervellione, Ester Maria Concetta Lombardo, Silvia Geraci, Calogero Iacolino

**Affiliations:** Department of Human and Social Sciences, University of Enna "Kore", Enna, Italy

**Keywords:** prenatal bonding, maternal–fetal attachment, paternal bonding, emotion regulation, infancy, early development, maternal mental health

## Abstract

**Introduction:**

Prenatal bonding is increasingly recognized as a foundational process for postnatal development, particularly in shaping infants’ emerging emotion regulation. This review aimed to synthesize empirical evidence on the association between prenatal bonding and early emotion regulation capacities in infancy and toddlerhood (0–36 months).

**Methods:**

Following PRISMA 2020 guidelines, PubMed, EBSCOhost, and Scopus were systematically searched for English-language studies published between 2015 and 2025. Eligible studies assessed prenatal bonding—primarily maternal, with limited paternal inclusion—and postnatal emotion regulation outcomes in children aged 0–36 months. Methodological quality was appraised narratively due to substantial heterogeneity in designs, measures, and outcomes; a structured narrative synthesis was therefore undertaken.

**Results:**

Fourteen studies met inclusion criteria; eleven constituted the primary synthesis set (prenatal measures with outcomes ≤ 36 months), and three were considered contextually. Across studies, higher-quality prenatal bonding—particularly in the maternal domain—was associated with more favourable early regulatory indicators, notably lower negative affectivity and greater soothability. Evidence for attentional regulation and broader socioemotional adjustment was promising but more variable. Maternal mental health and sociodemographic factors emerged as consistent moderators. Although only a minority of studies included fathers, preliminary findings suggest possible additive paternal contributions.

**Discussion:**

Findings underscore the developmental significance of prenatal bonding and the need for theory-driven, multimethod longitudinal research using developmentally sensitive measures and more diverse samples, including paternal cohorts.

## 1 Introduction

Prenatal bonding, defined as the emotional connection that a pregnant woman develops with her fetus, has gained increasing attention as a foundational process in early child development (Condon, 1993; Brandon et al., 2009). Although the terms prenatal bonding, maternal–fetal attachment, and maternal representations are sometimes used interchangeably, they are conceptually distinct. Prenatal bonding highlights the mother’s affective investment and emotional involvement toward the fetus—often expressed through behaviors such as talking to the unborn baby or imagining future caregiving roles (Condon, 1993). Maternal–fetal attachment encompasses broader emotional and cognitive aspects of the relationship, including expectations, fantasies, and protective behaviors (Siddiqui and Hägglöf, 2000). Maternal representations refer more specifically to internal working models or schemas about the unborn child and one’s future caregiving role (Slade, 2005). Clarifying these distinctions is essential for theoretical coherence and empirical rigor in perinatal research (Table 1).

**Table 1:** Summary of key distinctions among these constructs.

Term	Definition	Focus	Typical measures
Prenatal Bonding	Affective emotional connection that the mother feels toward the fetus.	Emotional investment; behaviors such as talking to or touching the belly.	MAAS (Condon, 1993); PAI (Müller, 1993); PPBS (Cuijlits et al., 2016)
Maternal–Fetal Attachment	Broader emotional and cognitive aspects of the mother–fetus relationship.	Protective behaviors; expectations and fantasies about the child.	MFAS (Cranley, 1981)
Maternal Representations	Internal models/schemas of the unborn child and the future caregiving role.	Narrative coherence; reflective functioning; anticipated caregiving.	Working Model of the Child Interview [WMCI; Slade (2005)]

While overlapping in some dimensions, these constructs have distinct theoretical emphases; in this review, PPBS is considered a prenatal bonding measure [development: Cuijlits et al. (2016) and use: de Waal et al. (2024)].

Historically rooted in attachment theory (Bowlby, 1969/1982), the concept of prenatal bonding has evolved to encompass emotional, cognitive, and behavioral components of the maternal–fetal relationship. This includes the mother’s emotional investment in the unborn child, caregiving intentions, and the imagined quality of the postnatal mother–infant relationship (Mikulincer and Shaver, 2005). The bonding process typically strengthens as pregnancy progresses and is thought to lay the foundation for postnatal attachment, caregiving behaviors, and early dyadic attunement (Alhusen et al., 2013; Maas et al., 2016). Indeed, higher prenatal bonding has been associated with greater maternal sensitivity postpartum and more favorable child socioemotional functioning (de Cock et al., 2016).

Infant emotion regulation (ER)—the capacity to modulate arousal, express affect, and recover from distress—emerges early in the postnatal period and is a core developmental competency (Gross and Thompson, 2007; Calkins and Hill, 2007). These early regulatory skills contribute to later social competence, behavioral adjustment, and psychological well-being (Cole et al., 2017; Eisenberg et al., 2010). In line with developmental models, we explicitly distinguish ER from temperament: temperament reflects biologically based predispositions (e.g., negative affectivity, surgency/effortful control), whereas ER refers to dynamic, often socially mediated processes that modulate affective states (Rothbart and Bates, 2006; Cole et al., 2017). Consistent with this distinction—and with the outcome hierarchy used in this review (see Section 2.4)—we differentiate primary ER indicators (e.g., soothability, distress recovery, attentional orienting/regulation), temperament proxies (e.g., negative affectivity, surgency/effortful control), and broader socioemotional adjustment (e.g., BITSEA competence (Briggs-Gowan and Carter, 2006); CBCL domains) (Achenbach and Rescorla, 2000). In parallel, maternal sensitivity, emotional availability, and the quality of dyadic interaction are recognized as critical environmental influences on the development of these capacities (Feldman, 2007; Tronick, 1989).

Recent developmental and neurobiological accounts suggest that the foundations of ER may be shaped even before birth. Intrauterine exposure to maternal affective states, hormonal patterns, and behavioral rhythms may influence fetal neurobehavioral development, thereby “scaffolding” early regulatory functions (Monk et al., 2019; Davis et al., 2019). Within this framework, prenatal bonding may operate indirectly—by promoting sensitive postnatal caregiving through more coherent maternal representations—and directly—by aligning maternal rhythms and expectations with the infant’s emerging regulation, potentially serving as a protective factor for early emotional competencies (Lebel and Deoni, 2018; Van den Bergh et al., 2020).

Prenatal bonding is shaped by a range of maternal psychological factors, including stress, anxiety, depression, adverse childhood experiences (ACEs), dispositional mindfulness, reflective functioning, and spiritual beliefs (Alhusen et al., 2013; Muzik et al., 2015; Slade, 2005; Gambin et al., 2021; Gheibi et al., 2020; Papini et al., 2022). For instance, maternal depression and anxiety have been linked to impaired bonding and less optimal infant outcomes (Grant et al., 2008; Henrichs et al., 2023), whereas higher maternal mindfulness and spiritual well-being are associated with stronger prenatal bonding and better dyadic coordination (Duncan et al., 2009; Malmir et al., 2023; de Waal et al., 2024).

Despite growing interest, few systematic reviews have focused specifically on the association between prenatal bonding and early emotion regulation. Existing syntheses more often examine maternal–fetal attachment in relation to postnatal bonding, caregiving, or general child outcomes (Barbu and Benga, 2024; Němcová et al., 2025; Tichelman et al., 2019; Bernal Rivas and Avello-Sáez, 2023), sometimes conflating constructs or lacking a focused developmental lens on self-regulatory competencies. Substantial heterogeneity in how prenatal bonding (e.g., MAAS, PAI, MFAS, PPBS) and infant outcomes (e.g., IBQ-R (Gartstein and Rothbart, 2003), BITSEA, CBCL; Global Rating Scales of Mother–Infant Interaction [GRS] Murray et al. (1996)) are operationalized further complicates cross-study comparisons and raises concerns about conceptual clarity and comparability. To ensure consistency across this literature, and acknowledging that some validated instruments tap both affective and representational facets, we use prenatal bonding as an umbrella term referring to studies assessing the emotional or representational connection between mother and fetus through validated measures.

Beyond individual caregiving factors, relational–ecological and group-analytic perspectives emphasize that bonding-related meanings and practices are culturally embedded—shaped by shared narratives, group norms, and implicit communicative rules within families and communities. Such interpersonal and cultural matrices can influence how caregivers construe prenatal experiences and anticipate early relational exchanges, with downstream implications for infants’ emerging self-regulatory competencies (Mannino and Giunta, 2015; Mannino et al., 2015). Positioning prenatal bonding within these broader matrices helps explain cross-context variability in bonding trajectories and early regulatory outcomes, complementing biological and dyadic mechanisms.

Notably, most empirical work centers on maternal bonding. Paternal measures are comparatively scarce, but where available they suggest potentially unique and additive influences on early socioemotional development. In this review we highlight such evidence where present and explicitly note gaps stemming from the underrepresentation of paternal cohorts.

### 1.1 Objectives of the review

This systematic review aims to:

- Synthesize empirical evidence on the association between prenatal bonding and emotion regulation in children aged 0–36 months.
- Examine the predictive role of prenatal bonding across clinical (e.g., maternal illness, high-risk samples) and non-clinical contexts, highlighting paternal findings where available.
- Evaluate how maternal psychological variables—such as depression, anxiety, stress, mindfulness, reflective functioning, adverse childhood experiences, and spirituality—mediate or moderate this association.
- Critically appraise methodological approaches, including instruments used to assess prenatal bonding (e.g., MAAS, MFAS, PAI, PPBS) and infant outcomes (e.g., IBQ-R, BITSEA, CBCL, GRS), and justify the use of prenatal bonding as an umbrella term when validated measures tap affective/representational connections.
- Identify limitations and gaps and outline theory-driven, multimethod, longitudinal research priorities using developmentally sensitive measures and more diverse samples, with the parallel goal of informing early preventive and clinical interventions to enhance maternal–infant emotional health.

## 2 Methods

This systematic review was conducted in accordance with the PRISMA 2020 guidelines for reporting systematic reviews of health and psychological interventions (Page et al., 2021). The review protocol was not publicly registered (e.g., in PROSPERO); however, all methodological steps—eligibility criteria, search strategy, screening, data extraction, quality appraisal, and synthesis—were specified *a priori* in an internal protocol and executed transparently. Given substantial heterogeneity in study designs, measures, and outcomes, a meta-analysis was not undertaken. Instead, we conducted a structured narrative synthesis following Popay et al. (2006).

The literature search and screening were conducted between May and June 2025 using electronic resources provided by the University Library System of the University of Enna “Kore.” The review team comprised four authors with predefined roles: B.C. coordinated the process (conceptualization, supervision, final synthesis); C.I. provided methodological oversight; E.M.C.L. and S.G. independently screened records and contributed to data extraction and narrative synthesis. Discrepancies at any stage were resolved by discussion and consensus among all authors.

### 2.1 Search strategy

We searched three databases: PubMed (*n* = 553), EBSCOhost—PsycINFO, PsycArticles, MEDLINE Complete, CINAHL, SocINDEX—(*n* = 427), and Scopus (*n* = 15), for a total of 995 records. The strategy combined controlled vocabulary (e.g., MeSH) and free-text terms:

(“prenatal bonding” OR “maternal-fetal attachment” OR “antenatal bonding” OR “maternal attachment to fetus”) AND (“emotion regulation” OR “self-regulation” OR “emotional development” OR “temperament” OR “socioemotional development”) AND (infant OR baby OR toddler OR child OR newborn OR offspring)

Limits: English language; publication years 2015–2025. Reference lists of included papers and relevant reviews were scanned narratively to contextualize findings (no additional database searches were added). Records were exported to Zotero for management and deduplication; no automation tools were used to screen records.

### 2.2 Eligibility criteria

- Inclusion criteria. Peer-reviewed empirical studies; human samples; infant/child outcomes assessed at 0–36 months; at least one prenatal bonding/attachment/representation measure (e.g., MAAS, PAI/PAI-R, PPBS, MFAS, WMCI); at least one postnatal outcome indexing emotion regulation or closely related domains; English-language publications, 2015–2025.
- Exclusion criteria. No eligible infant outcome or age range outside 0–36 months; absence of a prenatal bonding construct/measure; primarily medical or non-developmental focus; non-English publications.

### 2.3 Screening and study selection

After removing 126 duplicates, 869 records were screened by title/abstract by two independent reviewers. Twenty-nine full texts were assessed; 15 were excluded (no infant ER outcome, *n* = 7; no prenatal bonding construct/measure, *n* = 6; primarily medical focus, *n* = 2). Fourteen studies met the broad inclusion criteria and were retained.

**Figure 1:**
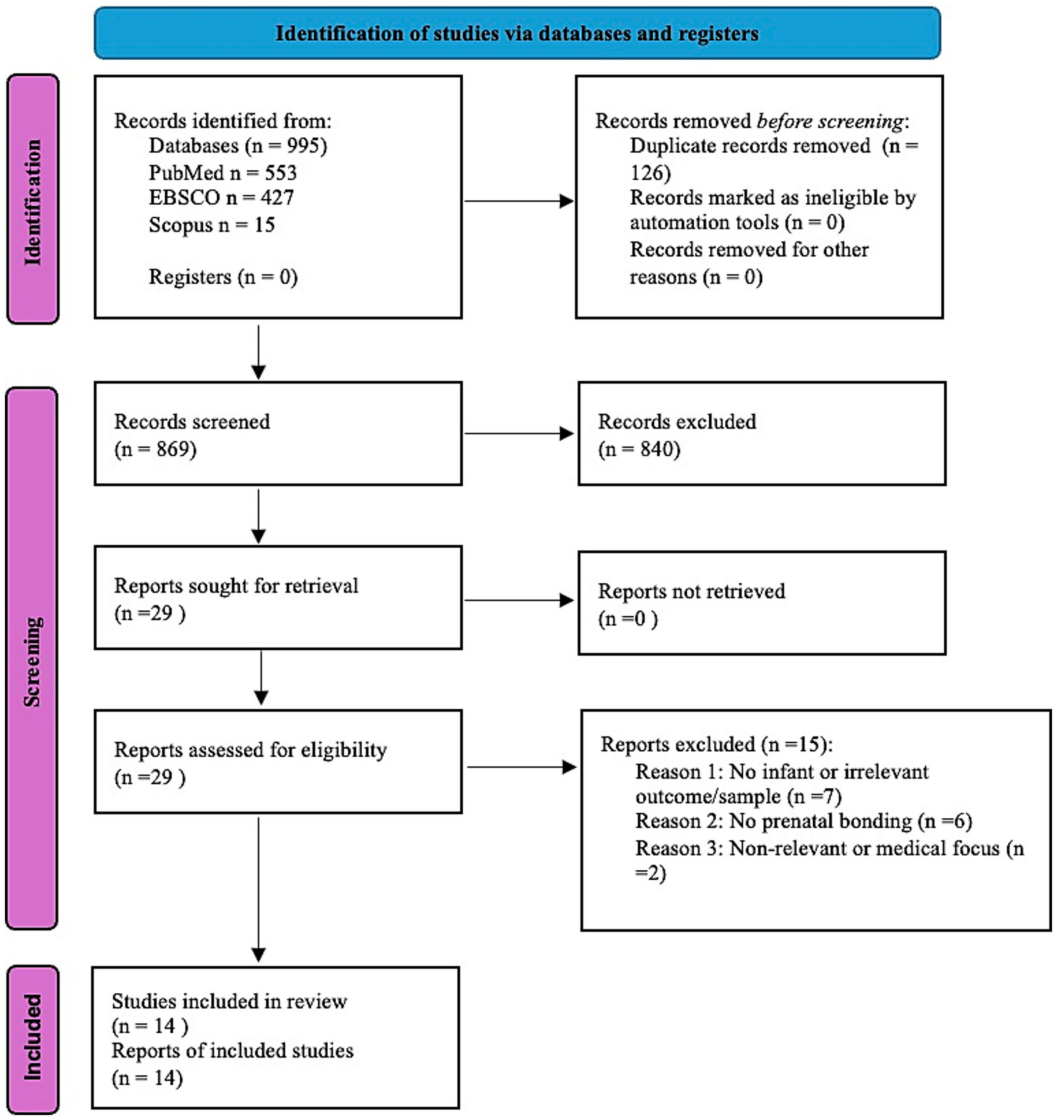
PRISMA 2020 flow diagram of study selection.

To enhance inferential precision, we specified *a priori* a primary synthesis set including studies that (i) assessed prenatal bonding/representations and (ii) reported postnatal outcomes ≤ 36 months; 11 studies met these criteria. Three additional studies were considered contextually (e.g., postnatal bonding only; no direct prenatal bonding measure; outcomes > 36 months) to inform interpretation but were not counted toward the primary synthesis. When multiple reports derived from the same cohort were identified, we retained the most comprehensive/least biased report for quantitative descriptors and treated companion papers as contextual evidence. The selection process is depicted in Figure 1 (PRISMA 2020 flow). Reasons for exclusion at full-text stage are summarized in Figure 1.

### 2.4 Data items and extraction

A structured codebook guided extraction by two reviewers. We recorded: bibliographic details; country; design (e.g., prospective longitudinal, cross-sectional, pilot/intervention); sample size and characteristics (e.g., risk status, education/socioeconomic status (SES) when reported); timing of assessments; prenatal predictor (instrument and construct class: prenatal bonding [e.g., MAAS, PAI/PAI-R, PPBS], maternal–fetal attachment [MFAS], maternal representations [e.g., WMCI]); presence of paternal antenatal measures (yes/no; instrument); outcomes (instrument, reporter, method: parent-report vs. observational/physiological); covariates/confounders; analytic approach (e.g., mediation/moderation). Disagreements were resolved by consensus; study authors were not contacted for additional data. Record management (search outputs, deduplication logs) was handled in Zotero.

To align with our conceptual framework (Section 2.5), each outcome was mapped *a priori* to one of three tiers: Primary Emotion Regulation (ER) (e.g., soothability/self-soothing, distress recovery, coder-rated regulatory behaviors, attentional orienting/regulation), Temperament proxies (e.g., negative affectivity, surgency/effortful control), and Broader socioemotional adjustment (e.g., BITSEA competence, CBCL internalizing/externalizing). When studies reported multiple outcomes, each was coded within its tier.

### 2.5 Operational definitions and outcome hierarchy

Predictor constructs were categorized as: (A) Prenatal Bonding (MAAS, PAI/PAI-R, PPBS), (B) Maternal–Fetal Attachment (MFAS), (C) Maternal Representations (WMCI). In line with developmental theory, we explicitly distinguished emotion regulation from temperament and treated temperament-based indices as proxies rather than primary ER endpoints. These a priori rules guided both extraction and synthesis.

### 2.6 Quality appraisal and narrative risk-of-bias evaluation

Given design heterogeneity, we did not apply a single numerical checklist (e.g., JBI, Newcastle–Ottawa Scale). Instead, we conducted a domain-based narrative appraisal adapted from ESRC guidance (Popay et al., 2006), organizing judgments along the following domains: Selection/Sampling Bias (sampling frame/representativeness; inclusion/exclusion transparency; recruitment/participation rates); Measurement Bias (validity/reliability of prenatal bonding instruments; clarity of outcome operationalization; assessment method—parent-report vs. observational/physiological; risk of common-method variance when the same reporter/instrument family assessed both predictor and outcome); Confounding/Model Specification (identification and control of maternal psychological variables—e.g., depression, anxiety, stress—sociodemographic factors—e.g., SES, education, partner support—and perinatal covariates; use of mediation/moderation models where appropriate); Attrition Bias (retention, differential dropout, missing-data handling); Reporting Bias (selective outcome reporting, analytic flexibility, congruence between stated aims and reported analyses). Each study was rated excellent/good/moderate based on the consistency of these features. Full appraisals with rationale are presented in Supplementary Table S1 (Quality Appraisal Summary).

### 2.7 Synthesis methods

Because predictors, measurement methods, and timings varied across studies, effect sizes were not pooled. We undertook a structured narrative synthesis by: (i) grouping studies by predictor category (A/B/C) and outcome tier (Primary ER / Temperament / Adjustment); (ii) integrating method considerations (reporter; observational vs. self-report) to interpret consistency; (iii) highlighting maternal psychological mediators/moderators; and (iv) summarizing paternal findings when available (noting that paternal data were sparse and did not drive the primary synthesis). A sensitivity decision defined *a priori* restricted the primary synthesis to outcomes assessed ≤ 36 months; studies with outcomes beyond this window were considered narratively only. Publication bias could not be formally assessed due to the absence of meta-analytic pooling; possible selective reporting was evaluated qualitatively within the reporting-bias domain. Interpretive claims prioritize Primary ER outcomes over temperament proxies and broader adjustment, consistent with the *a priori* hierarchy.

## 3 Results

### 3.1 Overview of included studies

Fourteen studies met the broad inclusion criteria; eleven comprised the primary synthesis set (prenatal bonding/representations with outcomes ≤ 36 months) and three were considered contextually (postnatal bonding only, no direct prenatal measure, or outcomes > 36 months). Unless otherwise noted, results refer to the primary synthesis set. Across designs, 9 were longitudinal, 4 cross-sectional, and 1 a pilot/intervention. Studies were conducted in South Korea, Finland, Brazil, China, the Netherlands, the United States, Switzerland, Australia, Turkey, and Italy, spanning Western and non-Western populations. Sample sizes ranged from *n* = 24 to *n* = 943.

Prenatal predictors were most commonly assessed with MAAS or PAI/PAI-R; PPBS was used in one study, MFAS in two, and WMCI (prenatal representations) in one. Infant outcomes were predominantly caregiver-reported (e.g., IBQ-R, BITSEA, CBCL, BRIEF-P), with one study including coder-rated observational interaction (Global Rating Scales, GRS). No study in the primary set included physiological indices of regulation. Consistent with the a priori framework (Section 2.5), distribution by predictor was: Prenatal bonding (A) = 8, Maternal–fetal attachment (B) = 2, Maternal representations (C) = 1. By outcome tier, Primary ER (e.g., soothability; attentional orienting/regulation) was represented by 2 studies; Temperament proxies by 3; and Broader socioemotional adjustment by 7 (with one study contributing to both proxy and adjustment tiers). Paternal antenatal indicators were sparse (e.g., PAAS alongside maternal MAAS in a single cohort) and did not drive the primary synthesis.

Overall, higher-quality prenatal bonding was associated with more favorable early regulatory indicators—most notably lower negative affectivity and greater soothability—with suggestive but less frequent evidence for attentional orienting/regulation. Associations with broader socioemotional adjustment tended to vary as a function of design, covariate control, and reporter. Several studies tested or discussed mediators/moderators, most consistently maternal mental health (depression/anxiety/stress) and sociodemographic context (e.g., education/SES, partner support); additional factors highlighted in the literature included dispositional mindfulness, reflective functioning, and spiritual well-being. A minority of studies reported null findings, suggesting potential influences of unmeasured contextual or interpersonal factors.

A sensitivity check restricting outcomes to ≤ 36 months yielded the same qualitative pattern; studies beyond this window were considered narratively and did not shape the primary conclusions. Common methodological limitations across the corpus included small sample sizes, reliance on maternal self-report, limited observational/multi-informant integration, and occasional attrition concerns, underscoring the need for larger, multi-method longitudinal research. For study-level characteristics and the construct-by-outcome mapping, see Tables 2, 3.

**Table 2:** Overview of included studies on prenatal bonding and infant emotion regulation.

Authors (year)	Country	Study design	Sample	Prenatal bonding measure	Infant emotion regulation measure
Bang et al. (2020)	South Korea	Longitudinal correlational cohort	97 pregnant women (initially 212; 54% dropout)	Maternal-Fetal Attachment Scale (*MFAS*) – Cranley	“What My Baby Is Like” scale (maternal report)
Rusanen et al. (2024) †	Finland	Longitudinal cohort	943 mothers and children (from pregnancy to age 2)	Postnatal bonding measure (no prenatal measure)	Brief Infant-Toddler Social and Emotional Assessment (*BITSEA*)
Rubin et al. (2022)	Brazil	Longitudinal population-based cohort	702 mother-infant dyads (pregnancy to 3 months)	*MFAS*	Bayley Scales of Infant Development – III (Social–Emotional)
Zhang et al. (2023)	China	Prospective longitudinal cohort	306 mother-infant pairs (to 6 months postpartum)	Maternal Antenatal Attachment Scale (*MAAS*)	Infant Behavior Questionnaire–Revised (*IBQ-R*), Very Short Form
de Cock et al. (2017)	Netherlands	Prospective longitudinal cohort	335 mothers and 261 fathers (to 24 months)	MAAS / Paternal Antenatal Attachment Scale (*PAAS*)	Behavior Rating Inventory of Executive Function – Preschool (*BRIEF-P*)
Calkins et al. (2024) †	USA	Longitudinal prospective cohort	150 families (to child age 3.5 years)	No direct bonding measure; proxies used (self-compassion, secure base)	*IBQ-R* – Negative Affectivity scale
Sancho-Rossignol et al. (2018)	Switzerland	Prospective longitudinal cohort	33 mother-infant dyads (pregnancy to 6 months)	*MAAS*	*IBQ-R* – Orienting/Regulation Scale
Branjerdporn et al. (2022)	Australia	Longitudinal correlational study	40 mother-infant dyads (12–24 months outcomes)	*MAAS*	Bayley Scales – Adaptive Behavior and Cognitive Scales
Lindstedt et al. (2024)	Finland	Prospective cohort (sub-study of STEPS)	97 families (with/without marital distress)	Working Model of the Child Interview (*WMCI*) – prenatal	*BITSEA* – Competence domain
Rossen et al. (2017) †	Australia	Population-based longitudinal cohort	372 pregnant women (three trimesters + 8w postpartum)	MAAS (T1-T3), MPAS at 8 weeks postpartum	Postnatal bonding only; infant emotional outcomes not assessed
Arguz Cildir et al. (2020)	Turkey	Longitudinal observational study	83 mother–child dyads (21–31 months outcomes)	Prenatal Attachment Inventory (*PAI*)	*BITSEA*; Ankara Developmental Screening Inventory (*ADSI*)
Bozicevic et al. (2022)	Italy	Observational longitudinal pilot study	24 mothers (11 with cancer history)	*PAI*	Global Rating Scales (*GRS*) – observational interaction
de Waal et al. (2024)	Netherlands	Prospective longitudinal cohort	408 pregnant women (to 12 months postpartum)	Pre- and Postnatal Bonding Scale (*PPBS*)	*IBQ-R*; infant social–emotional development (maternal report)
Henrichs et al. (2023)	Netherlands	Prospective longitudinal study	666 mothers and toddlers (to 28 months)	*MAAS* (32 weeks gestation)	Child behavior checklist (*CBCL*) – internalizing/externalizing

^†^Contextual study: not included in the primary synthesis (postnatal bonding only, no direct prenatal bonding measure, or outcomes >36 months).

**Table 3:** Construct–outcome mapping of included studies (primary synthesis set, *n* = 11).

Study (year)	Predictor category	Prenatal measure	Outcome tier	Infant outcome measure
Bang et al. (2020)	B (MFA)	*MFAS*	Proxy (temperament)	“What My Baby Is Like”
Rubin et al. (2022)	B (MFA)	*MFAS*	Adjustment	*Bayley-III* Social–Emotional
Zhang et al. (2023)	A (Bonding)	*MAAS*	Proxy (temperament facets)	*IBQ-R* (VSF)
de Cock et al. (2017)	A (Bonding; +*PAAS*)	*MAAS / PAAS*	Adjustment (EF)	*BRIEF-P*
Sancho-Rossignol et al. (2018)	A (Bonding)	*MAAS*	Primary ER (attentional regulation)	*IBQ-R* Orienting/Regulation
Branjerdporn et al. (2022)	A (Bonding)	*MAAS*	Adjustment	*Bayley* Adaptive/Cognitive
Lindstedt et al. (2024)	C (Representations)	*WMCI* (prenatal)	Adjustment	*BITSEA* Competence
Arguz Cildir et al. (2020)	A (Bonding)	*PAI*	Adjustment	*BITSEA*, *ADSI*
Bozicevic et al. (2022)	A (Bonding)	*PAI*	Primary ER (observational)	*GRS* (mother-infant interaction)
de Waal et al. (2024)	A (Bonding)	*PPBS*	Proxy and adjustment	*IBQ-R*; maternal report of socioemotional development
Henrichs et al. (2023)	A (Bonding)	*MAAS* (32w)	Adjustment	*CBCL* (28 m)

## 4 Discussion

### 4.1 Association between prenatal bonding and infant emotion regulation

Across the included studies, a recurring pattern indicates a meaningful association between higher-quality maternal prenatal bonding and more favorable infant regulatory profiles. Both longitudinal and cross-sectional designs linked stronger bonding—typically assessed via validated self-report tools such as the MAAS and PAI—to greater soothability, lower irritability/negative affectivity, and, in some cases, better socioemotional competence (Rubin et al., 2022; Henrichs et al., 2023; Arguz Cildir et al., 2020). Although observational corroboration was less frequent, available behavioral assessments converged with caregiver reports, suggesting that maternal affective investment during pregnancy relates to infants’ early regulatory capacities beyond maternal perceptions alone.

Continuity also appeared relevant: studies tracking bonding from pregnancy into the postpartum period suggested that more stable, sustained bonding was associated with more adaptive emotional trajectories (de Cock et al., 2016; Zhang et al., 2023), aligning with attachment-informed developmental models and underscoring continuity between prenatal representations and postnatal caregiving sensitivity. At the same time, emerging evidence points to bidirectionality: infant negative emotionality can shape parental perceptions of bonding and co-parenting quality (Calkins et al., 2024). Taken together, prenatal bonding is best conceived not as a fixed antecedent but as a dynamic construct embedded in a broader, reciprocal relational system. Given predominantly nonexperimental designs, these associations should not be interpreted as causal.

### 4.2 Dimension-specific summary

Associations were most consistent for primary ER indicators related to soothability/self-soothing and for temperament proxies such as reduced negative affectivity. Evidence for attentional orienting/regulation—a primary ER facet—was positive but less common, likely reflecting fewer studies directly targeting attentional processes in the first two years. Links with broader socioemotional adjustment (e.g., BITSEA competence; CBCL domains) varied as a function of design features, covariate control, and reporter. Notably, representational measures (e.g., prenatal WMCI) tended to relate more robustly to competence/adjustment than to discrete micro-indices of regulation, suggesting partially distinct explanatory pathways for affective bonding versus representational predictors.

### 4.3 Potential mechanisms

Converging developmental and neurobiological accounts suggest multiple, complementary pathways through which prenatal bonding may shape early ER:

- Maternal stress physiology during pregnancy (e.g., HPA axis signaling) may influence fetal neurobehavioral maturation, seeding nascent regulatory capacities;
- Coherent prenatal representations and higher reflective functioning may scaffold sensitive postnatal caregiving that supports infant regulation; and.
- Early dyadic synchrony/co-regulation may consolidate emerging regulatory strategies.

While consistent with theoretical models (e.g., Monk et al., 2019; Davis et al., 2019; Van den Bergh et al., 2020), these pathways were rarely tested directly in the primary set (e.g., via biomarkers or autonomic indices), rendering mechanistic inferences provisional. Consistent with our Results (Section 3.1), none of the primary-set studies incorporated physiological indices, underscoring that mechanistic claims should remain tentative.

### 4.4 Maternal psychological factors as mediators or moderators

Maternal psychological functioning emerged as a robust contextual layer modulating bonding–ER links. Symptoms of depression, anxiety, and stress were associated with diminished bonding and less optimal infant outcomes (Bozicevic et al., 2022; Sancho-Rossignol et al., 2018). Conversely, dispositional mindfulness—particularly non-judging—was linked to stronger bonding and fewer socioemotional difficulties (de Waal et al., 2024). Early adversity/trauma and unresolved attachment-related distress appeared detrimental to bonding, plausibly via reduced reflective functioning and greater emotion dysregulation (Branjerdporn et al., 2022; Slade, 2005). Several studies treated these variables as covariates or candidate mediators/moderators; taken together, the pattern supports perinatal mental health as a key leverage point for promoting both maternal–fetal bonding and early ER. In addition, emerging evidence on spiritual well-being suggests potential protective effects on bonding quality and dyadic coordination, warranting further investigation.

### 4.5 Sociodemographic and contextual influences (including paternal contributions)

Sociodemographic and ecological contexts shaped both prenatal bonding and its downstream correlates. Lower parental education and related stressors were associated with less coherent prenatal representations and lower child socioemotional competence (Lindstedt et al., 2024). Cultural practices such as Taekyo in Korea were linked to stronger bonding and favorable early outcomes (Bang et al., 2020). Relationship climate and partner support further modulated associations (Rubin et al., 2022; Zhang et al., 2023). Although most studies focused on mothers, the few that included paternal antenatal measures indicate potentially unique and additive paternal influences on early socioemotional development (de Cock et al., 2017; Lindstedt et al., 2024), with work highlighting paternal self-compassion as a possible protective resource within family dynamics (Calkins et al., 2024). Given the sparse paternal data, dedicated paternal cohorts and dyadic and triadic designs are needed. Overall, situating prenatal bonding within broader ecological models clarifies how individual, family, and cultural layers intersect to shape developmental trajectories.

## 5 Methodological and conceptual considerations

“Emotion regulation” was operationalized heterogeneously across the corpus. Some studies targeted primary ER (e.g., soothability, distress recovery, attentional orienting/regulation), while others used temperament proxies (e.g., negative affectivity, surgency/effortful control) or broader socioemotional adjustment (e.g., BITSEA competence, CBCL). As emphasized in this review, ER and temperament are conceptually distinct: temperament reflects biologically based predispositions, whereas ER refers to dynamic, often socially mediated processes for modulating affect (Rothbart and Bates, 2006; Cole et al., 2017). This heterogeneity complicates cross-study synthesis and underscores the need for theory-driven, developmentally sensitive measurement that can disaggregate reactivity, regulation, and adjustment.

Methodologically, heavy reliance on maternal self-report for both predictors and outcomes introduces shared-method variance and potential reporter bias. Future work should prioritize multi-method [questionnaires + observational coding + physiological indices such as cortisol or respiratory sinus arrhythmia (RSA)], multi-informant, and multi-timepoint designs to strengthen inference, alongside greater sample diversity (fathers/co-parents; under-represented sociodemographic groups; clinical/high-risk contexts) and harmonized measures to improve comparability. Protocol preregistration (e.g., PROSPERO/OSF) and a design-sensitive risk-of-bias framework—even when meta-analysis is not feasible—would increase transparency. Validated observational paradigms such as the Still-Face Paradigm (Tronick et al., 1978), the Emotional Availability Scales (Biringen, 2000), and structured play-based assessments can enhance ecological validity and capture behavioral manifestations of ER beyond questionnaires; where feasible, integrating autonomic and endocrine markers would allow more direct tests of hypothesized mechanisms.

## 6 Limitations and gaps in the literature

Despite encouraging findings, the current literature presents several gaps. First, most samples were low-risk, well-educated, and predominantly Western, limiting generalizability to underrepresented groups, including fathers/co-parents, same-sex couples, ethnic minorities, and families experiencing psychosocial adversity. Second, many studies relied heavily on maternal self-report for both predictors and outcomes, with sparse use of observational paradigms and minimal use of physiological indices of regulation in the primary set; this raises concerns about shared-method variance and constrains mechanistic inference. Third, construct and measurement heterogeneity—particularly the frequent use of temperament proxies in lieu of primary emotion regulation (ER) endpoints—complicates cross-study comparability. Fourth, attentional orienting/regulation in the first two years was under-assessed relative to affective facets (e.g., soothability, negative affectivity). Fifth, paternal prenatal bonding was rarely measured, leaving the potential unique and additive paternal contributions largely unexplored. Sixth, continuity beyond the prenatal phase—that is, the stability of bonding/representations into the postpartum and its linkage with caregiving behaviors—was inconsistently assessed, limiting inferences about developmental cascades. Finally, several studies had modest sample sizes and variable covariate control (e.g., maternal mental health, SES), and mediation/moderation tests were inconsistently applied.

There is a pressing need for integrative, theory-driven research that captures the interplay among biological, psychological, and sociocultural processes. Longitudinal and experimental designs—including randomized controlled trials—are warranted to test causal mechanisms and evaluate modifiable targets. Importantly, greater attention to clinical and high-risk populations (e.g., perinatal mood/anxiety disorders, medical complications, adolescent pregnancy, socioeconomic adversity) is essential to improve ecological validity and translational relevance.

## 7 General conclusions

This review identifies a consistent pattern linking higher maternal prenatal bonding to more favorable early indicators of infant ER—especially greater soothability and lower negative affectivity—while acknowledging construct and measurement heterogeneity. Associations were most robust when bonding was stable across the perinatal period, aligning with attachment-informed models that emphasize continuity from prenatal representations to postnatal caregiving sensitivity. At the same time, given predominantly nonexperimental designs, these associations should not be interpreted as causal.

The findings support integrative frameworks in which prenatal affective/representational processes contribute to early socioemotional development through maternal emotional availability, reflective functioning, and dyadic coordination. Clinically, the evidence underscores the potential value of assessing and supporting maternal–fetal bonding during pregnancy as a modifiable and developmentally consequential relational process. Methodologically, advancing the field will require clearer construct differentiation (ER vs. temperament), multi-method assessment, and more diverse samples—including fathers/co-parents and clinical/high-risk groups. By focusing specifically on ER as a developmental outcome, this review offers a targeted, clinically actionable perspective on prenatal bonding and lays the groundwork for more integrative, developmentally informed research.

## 8 Practical and clinical implications

From a clinical and preventive standpoint, several implications emerge:

Screening and monitoring. Brief, validated instruments (e.g., MAAS, PAI/PAI-R, PPBS) can be used during routine prenatal care to identify bonding difficulties and contextual risk (e.g., maternal distress), without pathologizing normative variability.

Targeted supports. Interventions that enhance maternal psychological resources—mindfulness- and compassion-based strategies, reflective functioning, and emotion-regulation skills—may strengthen prenatal representations and buffer the effects of distress. Where feasible, integrating observational feedback and bonding-focused components (e.g., guided imagery, emotional awareness practices, narrative elaboration) can complement self-report tools.

Family- and context-sensitive care. Given emerging signals for paternal contributions, father/co-parent inclusion (e.g., psychoeducation, partner-based mindfulness/communication modules) is recommended. Programs should be culturally responsive and linked to referral pathways for perinatal mental health services. Embedding these elements in childbirth education and routine prenatal programs can increase reach.

Stepped and integrated models. Embedding bonding-focused components within multidisciplinary, holistic perinatal care (obstetrics, mental health, primary care, community services) may facilitate early identification, low-burden supports, and timely escalation when needed. Enhancing maternal emotional availability is a promising clinical target to promote early relational health and optimal socioemotional development.

## 9 Limitations of the review

This review has several limitations. The protocol was not preregistered; however, all methodological steps were defined *a priori* and reported transparently. We restricted inclusion to English-language publications (2015–2025), which may introduce language bias. Given substantial heterogeneity in designs, measures, and timings, we did not perform a meta-analysis and instead undertook a structured narrative synthesis. Risk of bias was appraised narratively using a domain-based approach aligned with JBI/NOS domains (sampling/selection, measurement validity, attrition, and control of confounders), rather than a single numerical checklist, which may limit comparability with reviews using standardized tools.

Study selection and coding followed an a priori outcome hierarchy (primary ER, temperament proxies, broader socioemotional adjustment), but mapping heterogeneous outcomes to tiers necessarily involved judgment. Although double screening and a structured codebook were used, we did not contact authors for missing data and did not include grey literature; publication bias could not be formally assessed. Finally, mechanistic inferences are constrained by the limited use of physiological indices in the primary set and by the predominance of self-report measures in the included studies.

## 10 Future research directions

To strengthen the evidence base and its translational impact, future studies should:

- Adopt multi-method designs that combine questionnaires with validated observational paradigms (e.g., Still-Face, Emotional Availability, structured play) and physiological markers (e.g., cortisol, RSA), enabling tests of specific regulatory mechanisms.
- Broaden sampling frames to include underrepresented groups and clinical/high-risk populations (perinatal mood/anxiety disorders, medical complications, adolescent pregnancy, socioeconomic adversity), and to systematically incorporate fathers/co-parents via dyadic/triadic designs; explicitly include same-sex couples.
- Clarify constructs and harmonize measures, distinguishing ER from temperament and mapping outcomes to developmentally sensitive domains; where possible, employ shared core batteries to improve comparability and enable data pooling.
- Strengthen methodological transparency through preregistration (e.g., PROSPERO/OSF), prespecified analysis plans (including mediation/moderation), adequate power, and open-science practices (e.g., data/code sharing where permissible).
- Track bonding trajectories longitudinally from pregnancy through infancy, integrating candidate mediators (e.g., maternal sensitivity, dyadic synchrony, postnatal attachment) to delineate causal pathways and developmental cascades.
- Test causal mechanisms with randomized controlled trials targeting modifiable maternal processes (mindfulness, reflective functioning, emotion regulation) and family-level supports (partner involvement, relational interventions).
- Collectively, these steps will help delineate specific mechanisms linking prenatal bonding to early ER, enhance generalizability, and inform scalable interventions to promote early socioemotional health.

## Data Availability

The original contributions presented in the study are included in the article/Supplementary material, further inquiries can be directed to the corresponding author.

## Supplementary material

Supplementary Table S1 Quality appraisal summary for all included studies, organized by methodological domain (selection, measurement validity, attrition, and confounding control). The appendix provides descriptions of all psychometric and observational measures used across studies, including prenatal bonding and infant emotion-regulation instruments.

PRISMA 2020 Checklist Completed checklist reporting adherence to PRISMA 2020 guidelines, with references to sections in the manuscript.
