# Supplementary material for: Prenatal bonding and early emotion regulation in infancy and toddlerhood (0–36 months): a systematic review of developmental associations, psychological mediators, and contextual moderators
Source: Front Psychol. 2025 Nov 12;16:1700636. doi: 10.3389/fpsyg.2025.1700636 (PMC12647035; doi:10.3389/fpsyg.2025.1700636)
Supplement: PRISMA 2020 Checklist — Completed checklist reporting adherence to PRISMA 2020 guidelines, with references to sections in the manuscript. [file Data_Sheet_2.pdf]

## *Supplementary Material*

### 1. Supplementary Table S1 – Quality Appraisal Summary

| Authors (Year) | Country | Study Design | Inclusion Tier (Primary/ Contextual) | Sample | Prenatal Bonding Measure | Infant Emotion Regulation Measure | Main Findings | Limitations | Quality Assessment | Notes |
|---|---|---|---|---|---|---|---|---|---|---|
| Bang et al. (2020) | South Korea | Longitudinal cohort correlational study | Primary | 97 pregnant women (initially 212 recruited; 54% dropout) | Cranley's Maternal-Fetal Attachment Scale | Infant temperament assessed by "What My Baby Is Like" scale | Higher prenatal and postpartum maternal depression correlated significantly with more difficult infant temperament. Taekyo (traditional prenatal care) | High dropout rate (>50%), localized sample limits generalizability; small final sample size. | Good. | Clear longitudinal design; reliable, validated measures used; addressed potential dropout bias with incentives.<br><br>Emphasizes cultural specificity (taekyo) in interpreting prenatal bonding and depression effects. |

Domain-based quality appraisal aligned with JBI/NOS domains (selection/sampling, measurement validity, attrition, confounding

| | | | | | | | | | | |
|---|---|---|---|---|---|---|---|---|---|---|
| | | | | | | | practices significantly correlated with better maternal-fetal attachment and negatively correlated with postpartum depression at 6 months. Difficult infant temperament was significantly associated with higher colic incidence. | | | |
| Rusanen et al. (2024) † | Finland | Longitudinal cohort study | Contextual | 943 mothers and their | Representations of Unborn | Brief Infant-Toddler | Prenatal negative expectatio | High attrition rate at 24 | Excellent | Robust longitudinal design, large |

Domain-based quality appraisal aligned with JBI/NOS domains (selection/sampling, measurement validity, attrition, confounding

| | | | | | | | | | | |
|---|---|---|---|---|---|---|---|---|---|---|
| | | | | children, followed from third trimester pregnancy to age two | Baby (RUB-M) scale | Social and Emotional Assessment (BITSEA) | ns about caregiving and poor postnatal maternal bonding at 3 and 8 months significantly predicted greater social-emotional problems at 2 years. Positive maternal relationships and family atmosphere reduced these problems. Maternal depression and stress exacerbated risks but did not | months; relatively high socioeconomic and educational status of sample limits generalizability. Prenatal bonding measured through maternal expectations rather than standardized prenatal attachment tools. | | representative cohort, thorough statistical control of confounders (stress, depression, demographics).<br><br>Reinforces cumulative impact of prenatal and postnatal maternal bonding on child socio-emotional development, emphasizing preventive interventions targeting maternal bonding and psychosocial support. |

Domain-based quality appraisal aligned with JBI/NOS domains (selection/sampling, measurement validity, attrition, confounding

| | | | | | | | | | | |
|---|---|---|---|---|---|---|---|---|---|---|
| | | | | | | | eliminate the predictive power of bonding variables. | | | |
| Rubin et al. (2022) | Brazil | Population-based longitudinal cohort study | Primary | 702 mother-infant dyads assessed from pregnancy to 3 months postpartum | Maternal-Fetal Attachment Scale (MFAS) | Bayley Scales of Infant Development - Third Edition (BSID-III) Social-Emotional Scale | Higher maternal-fetal attachment significantly predicted better infant social-emotional development at 3 months (β = 0.14, p = .002), even controlling for maternal mental health and sociodemographic variables. | Data collected through maternal self-report may introduce response bias; small effect size (2.7% variance explained). | Excellent | Large, population-based cohort, rigorous statistical adjustments, validated tools.<br><br>Highlights the prenatal bond as a key preventive target for enhancing infant emotional outcomes, emphasizing early intervention during pregnancy. |

Domain-based quality appraisal aligned with JBI/NOS domains (selection/sampling, measurement validity, attrition, confounding

| | | | | | | | | | | |
|---|---|---|---|---|---|---|---|---|---|---|
| Zhang et al. (2023) | China | Prospective longitudinal cohort study | Primary | 306 mother-infant pairs from pregnancy to 6 months postpartum | Maternal Antenatal Attachment Scale (MAAS) | Infant Behavior Questionnaire–Revised (Very Short Form) | Higher maternal prenatal bonding positively correlated with beneficial gut microbiota (more Bifidobacterium, less Burkholderia) and improved infant temperament, particularly surgency and effortful control. Burkholderia abundance mediated the association between | Potential selection bias; reliance on maternal self-report; microbiota measurement only at birth (neonatal meconium). | Excellent | Robust methodological approach, comprehensive multivariate controls, novel biological mediators included.<br><br>Highlights innovative linkages between prenatal bonding, gut microbiota composition at birth, and early behavioral temperament, providing novel insights into biological mechanisms underlying prenatal bonding effects. |

Domain-based quality appraisal aligned with JBI/NOS domains (selection/sampling, measurement validity, attrition, confounding

| | | | | | | | | | | |
|---|---|---|---|---|---|---|---|---|---|---|
| | | | | | | | maternal prenatal bonding and infant temperament. | | | |
| de Cock et al. (2017) | Netherlands | Prospective longitudinal cohort study | Primary | 335 mothers, 261 fathers and their children, from 26 weeks pregnancy to 24 months postpartum | Maternal and Paternal Antenatal Attachment Scale (MAAS/ PAAS) | Behavior Rating Inventory of Executive Function–Preschool Version (BRIEF-P) | Poorer prenatal bonding predicted poorer postnatal bonding and higher parenting stress. Higher parenting stress significantly mediated the association between poor parental bonding and executive functionin | Reliance on parental self-report may introduce response bias; potential reverse causality in cross-sectional data between stress and child outcomes at 24 months. | Excellent | Strong methodological rigor, longitudinal follow-up, validated scales, inclusion of fathers, robust mediation analysis.<br><br>Highlights the importance of parental bonding and stress as predictors of early cognitive and emotional self-regulation, emphasizing both maternal and paternal roles. |

Domain-based quality appraisal aligned with JBI/NOS domains (selection/sampling, measurement validity, attrition, confounding

| | | | | | | | | | | |
|---|---|---|---|---|---|---|---|---|---|---|
| | | | | | | | g problems in toddlers. | | | |
| Calkins et al. (2024) † | United States | Longitudinal prospective cohort study | Contextual | 150 families (couples followed from pregnancy to child's age of 3.5 years) | Not directly measured (assessed prenatal parental self-compassion and secure base in interparental relationship as proxies) | Infant Behavior Questionnaire-Revised (IBQ-R) Negative Affectivity scale | Higher infant negative emotionality at 1 year predicted poorer interparental conflict at 3.5 years via maternal perceptions of lower quality coparenting at toddlerhood. These negative effects were mitigated by higher prenatal paternal | Limited generalizability due to predominantly White, cisgender, mixed-sex couple sample; reliance primarily on self-report measures; specific focus on negative emotional rather than broader emotional regulation. | Excellent | Strong methodological rigor, multi-method and multi-informant design, robust longitudinal follow-up with controls for confounders and baseline measures.<br><br>Highlights bidirectional influences within family systems, emphasizing the importance of prenatal resources (self-compassion and secure base) in buffering negative child |

Domain-based quality appraisal aligned with JBI/NOS domains (selection/sampling, measurement validity, attrition, confounding

| | | | | | | | | | | |
|---|---|---|---|---|---|---|---|---|---|---|
| | | | | | | | self-compassion and secure interparental base, demonstrating these resources as protective factors. | | | temperament impacts on family dynamics. |
| Sancho-Rossignol et al. (2018) | Switzerland | Prospective longitudinal cohort study | Primary | 33 mother-infant dyads followed from pregnancy to 6 months postpartum | Maternal Antenatal Attachment Scale (MAAS) | Infant Behavior Questionnaire-Revised (IBQ-R), Orienting/ Regulation Scale | Maternal childhood exposure to domestic violence associated with poorer prenatal attachment, increased maternal heart rate reactivity to infant crying stimuli, and poorer | Small sample size; reliance on maternal self-report for infant regulation; simplistic assessment of domestic violence exposure (binary yes/no). | Good | Methodologically sound with physiological measures enhancing validity, though limited by small sample and lack of detailed trauma history.<br><br>Emphasizes the importance of maternal adverse childhood experiences screening during pregnancy for |

Domain-based quality appraisal aligned with JBI/NOS domains (selection/sampling, measurement validity, attrition, confounding

| | | | | | | | | | | |
|---|---|---|---|---|---|---|---|---|---|---|
| | | | | | | | infant emotional regulation at 6 months. Maternal prenatal heart rate negatively correlated with fetal heart rate, indicating physiological interplay. | | | preventive interventions to improve maternal-fetal attachment and infant emotional outcomes. |
| Branjerdporn et al. (2022) | Australia | Prospective longitudinal correlational study | Primary | 40 mother-infant dyads recruited in pregnancy, with infant assessments at 12–24 months | Maternal Antenatal Attachment Scale (MAAS) | Bayley Scales of Infant and Toddler Development – Adaptive Behavior and Cognitive Scales | Higher maternal-fetal attachment (MAAS), secure adult attachment, and maternal well-being predicted better maternal reports of | Small sample size; maternal self-report may introduce bias; generalizability limited by homogeneous sample. | Moderate to good | Validated tools used; limited by sample size and observational breadth.<br><br>Highlights the effect of prenatal attachment and maternal psychological health on infant outcomes; perinatal loss |

Domain-based quality appraisal aligned with JBI/NOS domains (selection/sampling, measurement validity, attrition, confounding

| | | | | | | | | | | |
|---|---|---|---|---|---|---|---|---|---|---|
| | | | | | | | infant adaptive behavior. Infants of mothers with a history of perinatal loss had lower observed cognitive scores. | | | has distinct developmental implications. |
| Lindstedt et al. (2024) | Finland | Prospective longitudinal cohort study | Primary | Final sample: 97 families (44 with marital distress, 53 without); part of the STEPS sub-study; prenatal WMCI and 18-month | Working Model of the Child Interview (WMCI), prenatal version; coded as balanced or non-balanced | Brief Infant–Toddler Social and Emotional Assessment (BITSEA), *Competence* domain | Balanced prenatal representations in both parents were associated with higher infant social-emotional competence at 18 months. No significant | Subsample from a larger cohort; lack of observational infant data; reliance on parent-report for outcomes; families limited to those with or without marital distress, | Excellent | Prenatal WMCI interviews conducted by trained researchers with double coding; validated outcome measure; rigorous selection and longitudinal design.<br><br>This study highlights the unique and additive role of |

Domain-based quality appraisal aligned with JBI/NOS domains (selection/sampling, measurement validity, attrition, confounding

| | | | | | | | | | | |
|---|---|---|---|---|---|---|---|---|---|---|
| | | | | BITSEA data complete | | | associations were found with emotional or behavioral problems. Maternal representations showed slightly stronger effects than paternal ones. | potentially restricting generalizability. | | maternal and paternal prenatal representations in predicting toddlers' social-emotional competence. Supports early interventions targeting parental mental representations during pregnancy. |
| Rossen et al. (2017) † | Australia | Population-based longitudinal cohort study | Contextual | 372 pregnant women assessed in all trimesters and at 8 weeks postpartum | Maternal Antenatal Attachment Scale (MAAS), assessed at T1, T2, and T3 | Maternal Postnatal Attachment Scale (MPAS), assessed at 8 weeks postpartum | Maternal-fetal bonding increased significantly in both quality and intensity throughout pregnancy. Stronger antenatal bonding at | Postnatal outcome limited to maternal self-report; the MAAS and MPAS are not structurally identical, limiting comparability; observation | Excellent | Large sample, repeated measures across pregnancy and postpartum, validated instruments, and multivariate modeling.<br><br>Highlights the predictive value of antenatal bonding quality |

Domain-based quality appraisal aligned with JBI/NOS domains (selection/sampling, measurement validity, attrition, confounding

| | | | | | | | | | | |
|---|---|---|---|---|---|---|---|---|---|---|
| | | | | | | | all trimesters predicted stronger postnatal bonding at 8 weeks. Additional predictors of lower postnatal bonding included older maternal age, non-English speaking background, being a first-time mother, employment status, breastfeeding problems, and infant crying. | al data on infant behavior were not included. | | for early maternal-infant bonding. Suggests prenatal interventions could improve postnatal relationship outcomes, especially in at-risk mothers. |

Domain-based quality appraisal aligned with JBI/NOS domains (selection/sampling, measurement validity, attrition, confounding

| | | | | | | | | | | |
|---|---|---|---|---|---|---|---|---|---|---|
| Arguz Cildir, Ozbek, Topuzoglu, Orcin, & Janbakhishov, (2020) | Turkey | Longitudinal observational study | Primary | 83 mother-child dyads; maternal data collected at 28–40 weeks gestation; child outcomes at 21–31 months | Prenatal Attachment Inventory (instrument not explicitly named, but likely based on validated scales; confirm if needed) | Brief Infant-Toddler Social and Emotional Assessment (BITSEA), Ankara Developmental Screening Inventory (ADSI) | Higher prenatal attachment scores predicted better emotional and behavioral competence (BITSEA) and developmental outcomes (ADSI). Prenatal bonding was a stronger predictor of child development than maternal depression. | Sample size relatively small; reliance on maternal self-report; potential unmeasured confounders | Moderate to good | clear methodology, validated tools, longitudinal design<br><br>BITSEA and ADSI assessed at 21–31 months; maternal depression and anxiety also analyzed |
| Bozicevic et al., (2022) | Italy | Observational longitudin | Primary | 24 mothers (11 with | Prenatal Attachment | Global Rating Scales | No significant group | Small sample size, pilot nature, | Moderate | Clear design and aim, validated |

Domain-based quality appraisal aligned with JBI/NOS domains (selection/sampling, measurement validity, attrition, confounding

| | | | | | | | | | | |
|---|---|---|---|---|---|---|---|---|---|---|
| | | al pilot study | | cancer history, 13 without); infants assessed at 2–5 months postpartum | Inventory (PAI) | (GRS; observational measure of mother–infant interaction, including infant affect and engagement) | differences in prenatal attachment or maternal mood; however, mothers with a cancer history were more remote and less absorbed with their infants, who in turn showed fewer positive communicative signals. | reliance on observational data at a single postpartum time point | | tools, but underpowered.<br><br>Relevant to understanding prenatal attachment effects on early socio-emotional interaction quality in clinical samples |
| de Waal et al., 2024 | Netherlands | Prospective longitudinal cohort study | Primary | 408 Dutch pregnant women (M age = | Pre- and Postnatal Bonding Scale (PPBS) | Infant Behavior Questionnaire – Revised | Higher levels of the mindfulness facet | Self-reported measures; maternal perceptions | High | Clear longitudinal design, validated instruments, adequate sample |

Domain-based quality appraisal aligned with JBI/NOS domains (selection/sampling, measurement validity, attrition, confounding

| | | | | | | | | | | |
|---|---|---|---|---|---|---|---|---|---|---|
| | | | | 31.33, SD = 3.59), assessed at 20 and 28 weeks of gestation, and at 10 weeks, 6 and 12 months postpartum. | | (IBQ-R); measures of infant temperament and social-emotional development | "non-judging" during pregnancy were associated with stronger pre- and postnatal maternal bonding and with fewer infant social-emotional problems in the first year of life. Maternal bonding partially mediated the association between mindfulness and | may bias reports of infant outcomes; no observational data; homogeneous Dutch sample limits generalizability. | | size, statistical path analysis.<br><br>Highlights the protective role of maternal mindfulness (specifically non-judging) in enhancing early bonding and reducing infant emotional problems; suggests potential for mindfulness-based interventions during pregnancy. |

Domain-based quality appraisal aligned with JBI/NOS domains (selection/sampling, measurement validity, attrition, confounding

| | | | | | | | | | | |
|---|---|---|---|---|---|---|---|---|---|---|
| | | | | | | | infant outcomes. | | | |
| Henrichs et al., 2023 | Netherlands | Prospective longitudinal study; secondary data analysis | Primary | 666 Dutch mothers and their toddlers (data collected at 24 and 32 weeks gestation, 6 weeks, 6 months, and 28 months postpartum) | Maternal Antenatal Attachment Scale (MAAS) – assessed at 32 weeks gestation | Child Behavior Checklist (CBCL) – internalizing and externalizing problems at 28 months | Maternal prenatal and postnatal bonding mediated the association between maternal prenatal anxiety and child externalizing problems. Only postnatal bonding (at 6 months) mediated the link between prenatal depression and child internalizi | Observational measures were not used (self-report only) No clinical diagnoses of maternal distress Attrition may have biased results | Moderate to high | Longitudinal design, standardized tools, large sample; some reliance on self-report.<br><br>Highlights the importance of both maternal distress and bonding in early development; supports early interventions targeting both prenatal distress and maternal bonding quality. |

Domain-based quality appraisal aligned with JBI/NOS domains (selection/sampling, measurement validity, attrition, confounding

| | | | | | | | | | | |
|---|---|---|---|---|---|---|---|---|---|---|
| | | | | | | | ng problems. Postnatal bonding was a more consistent mediator than prenatal bonding. | | | |

## 2. Appendix – Measures Used in Included Studies

***Descriptions are provided for all psychometric and observational tools used across the 14 included studies, covering both prenatal bonding and infant emotion regulation measures.***

### 2.1 Prenatal Bonding Measures

- **Maternal-Fetal Attachment Scale (MFAS) –** Measures maternal behaviors and attitudes toward the fetus (Cranley, 1981).

Domain-based quality appraisal aligned with JBI/NOS domains (selection/sampling, measurement validity, attrition, confounding

- **Maternal Antenatal Attachment Scale (MAAS) –** Assesses maternal emotional attachment to the unborn child, including quality and intensity (Condon, 1993).
- **Paternal Antenatal Attachment Scale (PAAS) –** Adapted version of the MAAS for fathers.
- **Representations of Unborn Baby – Maternal (RUB-M) –** Evaluates maternal cognitive and emotional representations of the fetus.
- **Working Model of the Child Interview – Prenatal Version (WMCI) –** Semi-structured interview coding caregiver representations as balanced or non-balanced.
- **Prenatal Attachment Inventory (PAI) –** Self-report measure assessing thoughts, feelings, and behaviors toward the unborn baby (Müller, 1993).
- **Pre- and Postnatal Bonding Scale (PPBS) –** Measures bonding in both prenatal and postnatal periods.
- **Proxy indicators –** In one study, prenatal self-compassion and perceived secure base in the interparental relationship were used as indirect measures.

### 2.2 Infant Emotion Regulation Measures

- **Infant Behavior Questionnaire–Revised (IBQ-R) –** Parent-report questionnaire assessing infant temperament, including negative affectivity and regulatory capacity (Gartstein & Rothbart, 2003).
- **Brief Infant–Toddler Social and Emotional Assessment (BITSEA) –** Screening tool for social–emotional problems and competence in young children.
- **Bayley Scales of Infant and Toddler Development – Third Edition (BSID-III) –** Includes social–emotional and cognitive scales for developmental assessment.

Domain-based quality appraisal aligned with JBI/NOS domains (selection/sampling, measurement validity, attrition, confounding

- **Behavior Rating Inventory of Executive Function – Preschool Version (BRIEF-P) –** Measures executive function behaviors in preschool-aged children.
- **Child Behavior Checklist (CBCL) –** Assesses behavioral and emotional problems in children, including internalizing and externalizing behaviors.
- **Global Rating Scales (GRS) –** Observational coding of dyadic interaction quality
- **Maternal Postnatal Attachment Scale (MPAS) –** Evaluates postnatal maternal attachment.
- **Ankara Developmental Screening Inventory (ADSI) –** Developmental screening tool validated in Turkish populations.
- **"What My Baby Is Like" Scale –** Parent-report measure of infant temperament.

Domain-based quality appraisal aligned with JBI/NOS domains (selection/sampling, measurement validity, attrition, confounding
